# Pleiotropic Modulation of Chitooligosaccharides on Inflammatory Signaling in LPS-Induced Macrophages

**DOI:** 10.3390/polym15071613

**Published:** 2023-03-23

**Authors:** Wentong Hao, Kecheng Li, Song Liu, Huahua Yu, Pengcheng Li, Ronge Xing

**Affiliations:** 1 CAS and Shandong Province Key Laboratory of Experimental Marine Biology, Center for Ocean Mega-Science, Institute of Oceanology, Chinese Academy of Sciences, Qingdao 266071, China; haowentong0723@163.com (W.H.); sliu@qdio.ac.cn (S.L.); yuhuahua@qdio.ac.cn (H.Y.); pcli@qdio.ac.cn (P.L.); 2 Laboratory for Marine Drugs and Bioproducts, Pilot National Laboratory for Marine Science and Technology (Qingdao), No. 1 Wenhai Road, Qingdao 266237, China; 3 University of Chinese Academy of Sciences, Beijing 100049, China

**Keywords:** chitooligosaccharides, anti-inflammatory mechanism, RNA-seq, inflammatory signaling pathway

## Abstract

Chitooligosaccharide (COS) is a green and non-toxic cationic carbohydrate that has attracted wide attention in recent years due to its anti-inflammatory activity. However, the anti-inflammatory mechanism of COS remains unclear. In this study, RNA-seq was used to investigate the integrated response of COS to LPS-induced damage in macrophages. The results showed that the experimental group with COS had 2570 genes with significant differences compared to the model group, and that these genes were more enriched in inflammatory and immune pathways. The KEGG results showed that COS induces the pleiotropic modulation of classical inflammatory pathways, such as the Toll-like receptor signaling pathway, NF-κB, MAPK, etc. Based on the RNA-seq data and the RT-qPCR, as well as the WB validation, COS can significantly upregulate the expression of membrane receptors, such as Tlr4, Tlr5, and MR, and significantly inhibits the phosphorylation of several important proteins, such as IκB and JNK. Overall, this study offers deep insights into the anti-inflammatory mechanism and lays the foundation for the early application of COS as an anti-inflammatory drug.

## 1. Introduction

Chitosan is a cationic polymer obtained from the deacetylation of chitin, the world’s most abundant natural polymer other than cellulose. It is the only cationic polysaccharide in nature. The presence of free amino groups allows chitosan to display many excellent properties, such as antibacterial activity, antioxidant activity, and antitumor activity. Furthermore, chitosan can be used in a variety of applications, such as surgical sutures [1,2,3,4]. However, the high molecular weight and viscosity of chitosan make it insoluble in most reagents and only soluble in acids [5]. These properties greatly hinder its application, especially in the pharmaceutical and medical fields. Chitooligosaccharide (COS) is the product obtained by the chemical or enzymatic degradation of chitosan. Compared to chitosan, COS has the characteristics of low molecular weight (degree of polymerization (DP) = 2 − 20), water solubility, and better biocompatibility. Furthermore, similar to chitosan, COS still has significant biological activities, such as antimicrobial activity [6,7], plant-growth-promoting activity [8], anti-inflammatory activity, anti-angiogenesis activity [9], etc. As clinical requirements for anti-inflammatory drugs have become more stringent, the anti-inflammatory and immune activities of COS have received increasing interest in recent years. For example, COS has been shown to reduce liver injury due to excessive inflammation or bursts of reactive oxygen species [10,11,12]. Furthermore, COS can alleviate enteritis damage in mice or turbot [13,14]. There is also some research suggesting that COS could modulate macrophage polarization in mice or blunt-snout bream [15,16]. Our previous work demonstrated the anti-inflammatory activity of COS in vitro and in vivo and found that COS with a degree of acetylation (DA) = 12% was the best. Although the anti-inflammatory activity of COS has been reported in various cells, such as RAW264.7 cells [17], THP-1 cells [18], and various animals [14,19,20], the anti-inflammatory mechanism of COS remains unclear. Most of the reports selected key proteins on typical inflammatory pathways for validation, such as P65, Akt, and PI3k, which are frequently detected proteins. The expression of these proteins is also used to speculate as to whether the anti-inflammatory activity of COS is involved in the relevant signaling pathways [21,22,23,24]. However, this type of validation is not sufficiently systematic and the workload is significant if we try to study the changes in all the intracellular cytokines and pathways after COS treatment.

Transcriptome is the collection of all the transcription products in a cell or organism under certain physiological conditions. It is the link between the genome (genetic information) and the proteome (biological function) and the main tool for studying gene expression. Transcriptome sequencing utilizes a new high-throughput sequencing platform to sequence genomic cDNA. The expression of different mRNAs is calculated by counting the number of relevant reads. Furthermore, the structure and expression levels of transcripts are analyzed to provide the most comprehensive transcriptome information. The transcriptome sequencing has evolved rapidly in recent years, from hybridization-based approaches or sequence-based approaches that are difficult to quantify and normalize to novel high-throughput DNA sequencing methods (RNA-seq). The RNA-seq method has been applied to many species, such as *Arabidopsis* [25], mice [26], and yeast [27], and has been widely used in basic research, clinical diagnosis, and drug development. The RNA-Seq has clear advantages over existing approaches. For example, RNA-Seq is not limited to detecting transcripts corresponding to existing genomic sequences and has been proven to have a very low background signal, as well as high accuracy [28]. At the same time, the amount of information that RNA-seq can offer is large. Additionally, RNA-seq can greatly reduce the time required for traditional experimental manipulations to investigate specific mechanisms.

Based on the previous work of our group [29], a COS (DA = 12%) with the best anti-inflammatory activity was selected to treat LPS-induced RAW 264.7 cells. Next, RNA-seq was performed on the control, model, and experimental groups, respectively. This study is the first to systematically analyze the anti-inflammatory mechanism of COS with the help of RNA-seq. Furthermore, we confirmed the pleiotropic modulation of COS on macrophages based on the validation of existing pathways. This lays the foundation for COS to quickly become a novel anti-inflammatory drug.

## 2. Materials and Methods

### 2.1. Reagents

The COS (DP = 2 − 6, DA = 12%) was purchased from Qingdao Yunzhou Biochemistry Co. (Qingdao, China). Fetal bovine serum (FBS) was purchased from Gibco (Carlsbad, CA, USA). RPMI 1640 medium, penicillin, and streptomycin were purchased from HyClone (Logan, UT, USA). Toll-like receptor 4 (Tlr4) inhibitor, Resatorvid (TAK-242), was purchased from MCE (Monmouth Junction, USA). Antibodies used in WB were purchased from Cell Signaling Technology (Boston, MA, USA). All other chemicals and reagents were of analytical grade.

### 2.2. Experimental Design

Macrophage RAW 264.7 cell lines were purchased from the Type Culture Collection of the Chinese Academy of Sciences (Shanghai, China). Cells were cultured in RPMI 1640 medium supplemented with 10% FBS, 1% double antibiotics (penicillin and streptomycin) in a humidified atmosphere with 5% CO_2_ at 37 °C. The experimental design is as follows: CK group (negative control, treated with only RPMI 1640 medium), LPS group (model group, treated with 1 μg/mL LPS), and EG group (experimental group, treated with 1 μg/mL LPS and 600 μg/mL COS). The concentration used in this experiment was determined based on our previous work [29]. Three biological parallels were set up for each group. The cells were inoculated into 6-well plates at a density of 1 × 10^5^ cells/mL and cultured for 24 h. The medium was then changed according to the grouping set above and incubated for another 24 h. Next, the supernatant was discarded and cells in each well were lysed for RNA extraction.

### 2.3. RNA-seq and Data Analysis

Total cellar RNA of each group was extracted using the E.Z.N.A total RNA kit II (Omega, Guangzhou, China). After the samples were tested, eukaryotic mRNA was enriched with magnetic beads of Oligo (dT) and purified and fragmented into small fragments by adding fragmentation buffer. The cleaved RNA fragments were reverse-transcribed into cDNAs. Next, fragment-size selection was performed with AMPure XP beads. Finally, PCR enrichment was performed to obtain the final cDNA library. After the libraries were constructed, the insert size and effective concentration of the libraries were tested. Different libraries were pooled according to the target downstream data volume and sequenced on the machine.

### 2.4. Real-Time qPCR (RT-qPCR) and Western Blotting (WB)

For RT-qPCR, the RNA was extracted using the E.Z.N.A total RNA kit II and quantified with Nanodrop 2000 for subsequent experiment. The RNA was reversed into DNA using the Evo M-MLV Reversal Kit. The RT-qPCR was performed using this DNA as a template by the SYBR Green Pro Taq HS Premix qPCR Kit amplification to detect gene-expression levels. The primer sequences for RT-qPCR were shown in Table 1.

For WB, cells were fully lysed on ice for 30 min using a cell-lysis solution containing protease inhibitor and phosphatase inhibitor. The extracted proteins were separated by 8–12% SDS-PAGE and blotted on PVDF membranes. The membranes were then blocked, incubated with corresponding antibodies, and subjected to ECL color development. Finally, semi-quantitative analysis of the proteins was performed by image J software. For RT-qPCR and WB experiments, at Least three biological parallels were set up for each group.

### 2.5. Statistical Analysis

Data in each experiment were analyzed using SPSS software. Furthermore, the data were presented as the mean ± SD, followed by Duncan’s multiple-range tests. Differences were statistically significant if *p* < 0.05.

## 3. Results

### 3.1. RNA-seq Data-Quality Assessment

In order to ensure the accuracy of the RNA-seq, a quality assessment was performed on the extracted RNA samples. For example, the original sequences contained reads with junction and low quality. The junction sequences needed to be removed. The reads with low quality (for which the average quality value of bases was less than 20) and N (meaning that the information for the bases could not be determined) of more than 5 needed to be filtered out. Subsequently, the clean reads that could be used for the subsequent analysis were obtained. The information on the raw reads, clean reads, Q20, etc., for each sample is summarized in Table 2. Additionally, Q20 and Q30 represent the base quality. The higher base-quality value indicates a more reliable base identification and a lower possibility of base-error measurement. For example, for a base-quality value of Q20, 1 out of 100 bases are identified incorrectly. For a base-quality value of Q30, 1 out of 1000 bases are identified incorrectly. In this experiment, the percentage of Q30 base was 90.54% or more.

**Figure 1:**
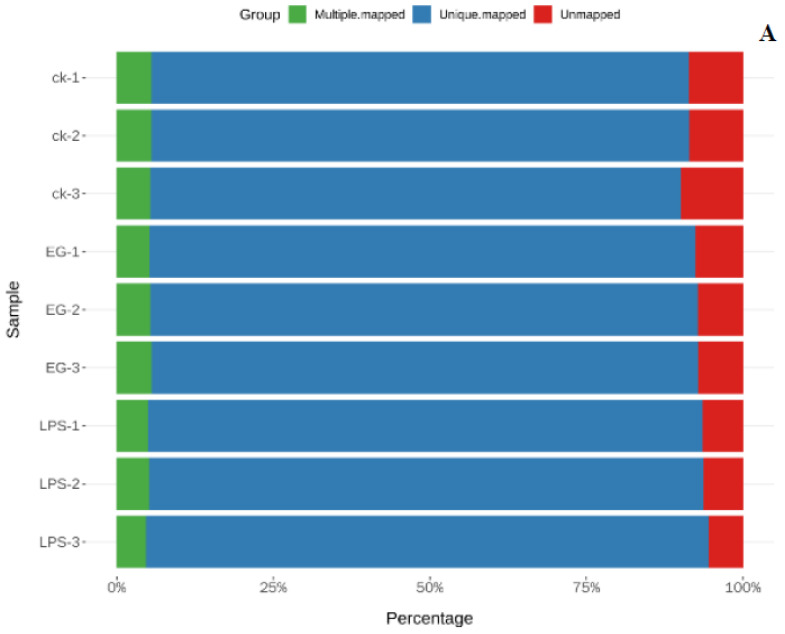

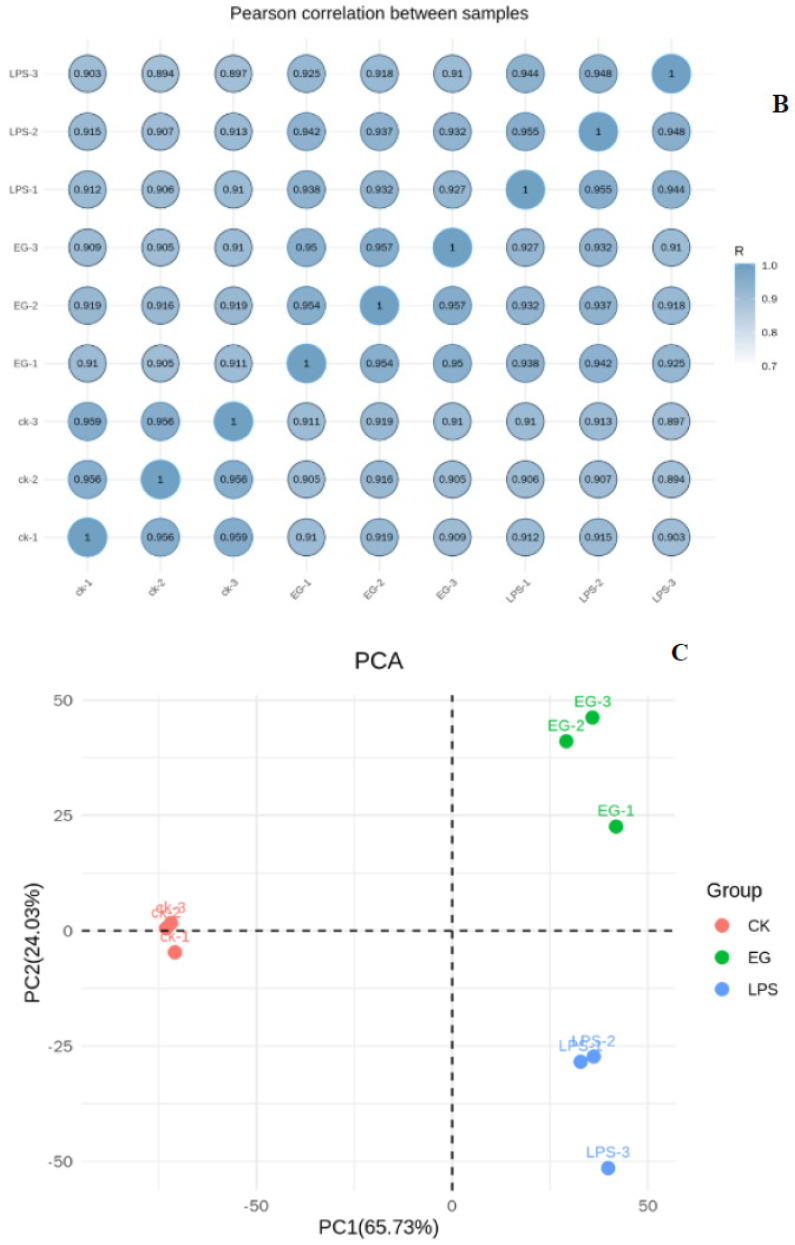
RNA-seq data–quality–assessment results. (**A**): Reads vs. reference genome comparison. (**B**): Inter–sample–correlation–coefficient heat map. (**C**): Two–dimensional principal−component–analysis plots.

After screening out the high-quality sequences, we used HISAT2 software to match the clean reads with the specified genome to obtain information as to its position on the reference genome. The comparison is shown in Figure 1A. More than 90% of the data can be located on the reference genome. The correlation of the gene-expression levels between samples is an important indicator to test the reliability of the experiment and the reasonability of the sample selection. The closer the correlation coefficient is to 1, the greater the similarity between the expression patterns of the different samples. It was generally required that the R between biological replicate samples should be at least greater than 0.8. The results of the gene-expression-correlation analysis on all the samples in this experiment are shown in Figure 1B. All the correlation coefficients were greater than 0.894. Subsequently, principal component analysis (PCA) was used to assess the differences between the groups and the biological reproducibility of the samples within the groups. In the PCA score plots, as shown in Figure 1C, there was a significant dispersion of gene-expression profiles between CK, LPS, and EG. The parallel samples from each group were clustered together, indicating the reliability and stability of the data. Therefore, the accuracy and reproducibility of the samples from this transcriptome experiment were verified. The screened data could therefore still be used to construct libraries and perform sequencing.

### 3.2. Differentially Expressed Genes (DEGs) Analysis

**Figure 2:**
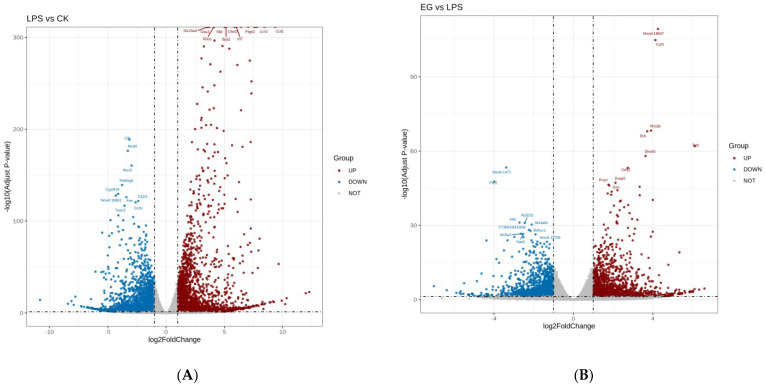
The volcano plots of LPS/CK and EG/LPS. (**A**): The volcano plots of LPS/CK. (**B**): The volcano plots of EG/LPS. A dot represents a gene. The gray area is for no significant difference The red dot represents a significant upregulated gene. The blue dot represents a significant downregulated gene. The larger the adjusted *p*−value, the more significant it is. The larger the |log2foldchange|, the greater the fold of difference in gene expression.

The differential gene analysis in RNA-seq is an independent statistical hypothesis test for many genes. It features the problem of a high overall rate of false positives. Therefore, we corrected the *p*-value obtained from the original hypothesis test. We used padj (adjusted *p*-value) < 0.05 and |log2foldchange| > 1 as the criteria for the significance of the differences. The DEGs analysis identified 5186 highly statistically significant genes in the LPS group, among which 2525 genes were strongly upregulated and 2661 were strongly downregulated. Furthermore, there were 2570 highly statistically significant genes in the EG/LPS, among which 1239 genes were strongly upregulated and 1331 were strongly downregulated. The volcano plots were drawn for the rapid identification of the genes with statistical significance (as shown in Figure 2). Combining our previous results [29] with the NO secretion by the cells used in this experiment (as shown in Figure S1), it can be concluded that the EG group has good anti-inflammatory activity. We subsequently focused on analyzing the RNA-seq results of the EG/LPS. The volcano plot of the EG/LPS showed that the addition of the COS affected the expression of massive genes in the mouse-macrophage-injury model. These DEGs mostly regulated the expression of inflammatory and immune-related proteins, such as Cd14, Tnf, Irak4, Cxcl2, Ccl4, Il-1r1, Tlr4, Cd40, and Cxcl3. For example, the EG group was able to significantly upregulate the expression of the Cxcl3 gene, which specifically moves chemotactic lymphocytes and monocytes towards the site of inflammation and enhances innate immunity. In addition, the EG group upregulated the expression of the intercellular adhesion factor. Some apoptosis-related genes were also significantly changed, such as Bcl2 and Cflar. Our next analysis was based on these DEGs and aimed to explore the anti-inflammatory mechanism of COS.

### 3.3. RT-qPCR

**Figure 3:**
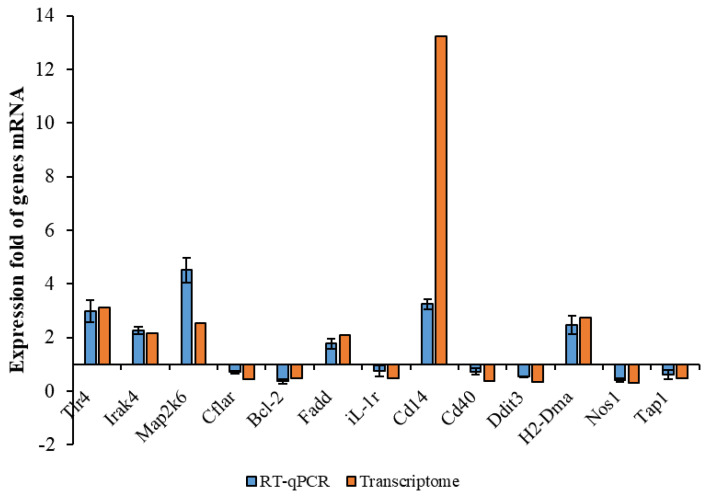
Comparison between transcriptome and RT−qPCR of some DEGs.

The data given in the RNA-Seq were used as a quantitative reference. The analysis results would have been affected by changes in parameters. Therefore, RT-qPCR validation was needed. Thirteen genes with large significant differences were selected randomly, six of which were selected from upregulated genes and seven from downregulated genes. These genes included inflammation-associated Tlr4, iL-1r, Cd40, apoptosis-associated Bcl-2, phagocytosis-associated Tap-1, H2-Dma, etc. Their gene-expression levels were examined using RT-qPCR and compared with the transcriptome results. As shown in Figure 3, the trends of the transcriptome results and RT-qPCR results were consistent, except for very few genes with slightly different expression ploidy. These results indicated that the transcriptome results were plausible. Based on this, we were able to the perform functional annotation and an enrichment analysis of the DEGs given by the RNA-seq.

### 3.4. Functional Annotation and Enrichment Analysis of DEGs

**Figure 4:**
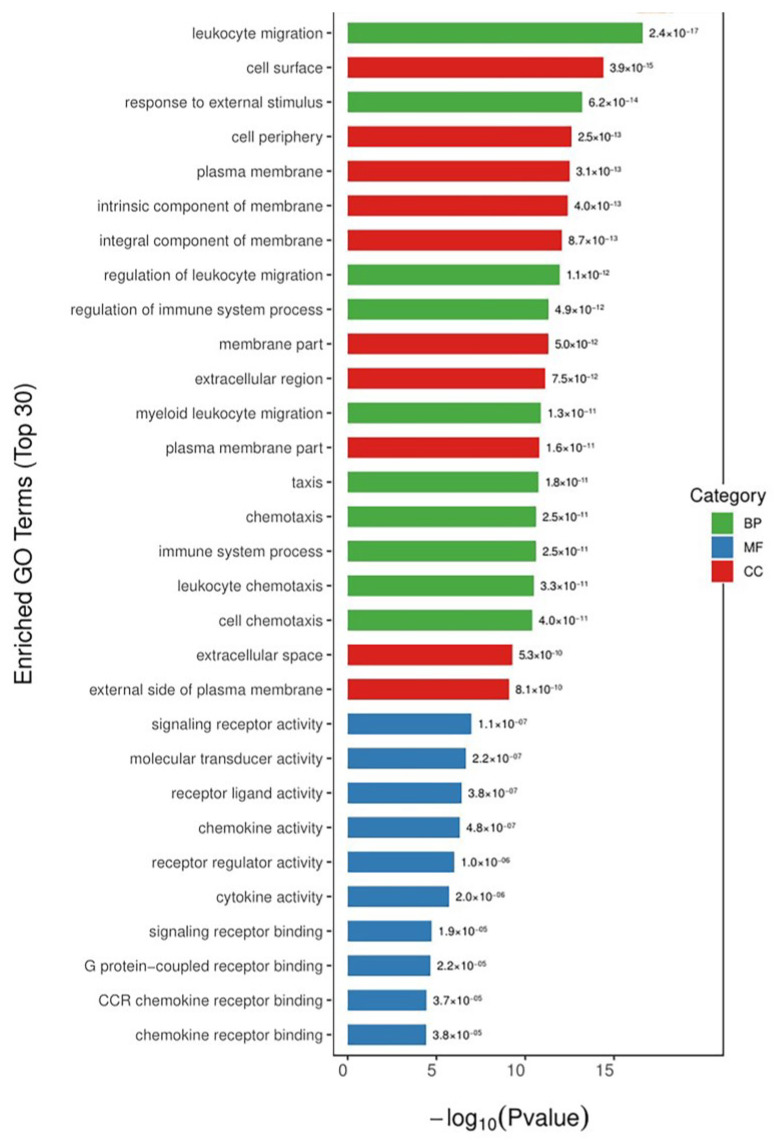
GO enrichment of DGEs. The chart was constructed by plotting the *p*-value on the *x*-axis and entries are listed in ascending order of *p*-values. Green indicates biological processes. Blue indicates molecular functions. Red indicates cellular components.

To gain more insight into the mechanism of the alleviating effect of COS on LPS-induced inflammation, the biological-function-annotation and -enrichment analyses of the differentially expressed genes screened previously were performed. The functional-enrichment analysis of the DEGs was performed by comparing all the DEGs with the annotated results of the genomes of the reference species according to the gene ontology (GO) functions against each other. The significance of the differences was derived by Fisher’s exact test to find the functional categories in which all the differentially expressed proteins were enriched (*p* value < 0.05). The results of the functional-enrichment analysis were used to directly reveal the overall functional enrichment characteristics of all the DEGs. The GO is a standardized functional classification system that describes the properties of genes and gene products in an organism in three ways: biological processes (BPs), molecular function (MF), and cellular components (CCs). We selected the 10 most significant entries under each category and present them in Figure 4. The DEGs induced by the EG group treated with COS were mainly involved in BPs, such as the migration and regulation of leukocytes, responses to external stimuli, the regulation of immune-system processes, and inflammatory responses; in MF, they were mainly involved in signal-receptor activity, molecular-transducer activity, receptor-ligand activity, chemokine activity, receptor regulatory activity, cytokine activity, and signal-receptor binding; in CC, they were mainly concentrated in the cell surface, cell periphery, plasma membrane, and other membrane components. The GO enrichment results showed that the differential genes in the EG group were indeed mostly enriched for inflammation and immune response, involving both signaling receptors and immune factor activities, as well as, potentially, the activity of membrane receptors.

**Figure 5:**
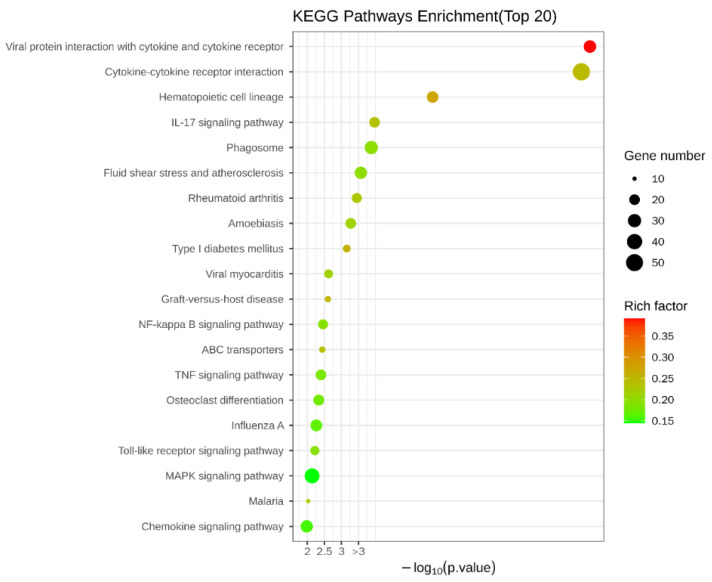
KEGG enrichment of DEGs. The size of the Rich factor is indicated by the color of the dots and the larger the value, the closer the color to red. The number of DEGs in each pathway is indicated by the size of the scatter.

To explain what specifically changed with the addition of COS compared to the injury-model group with LPS alone, the KEGG database was also analyzed. The results of the KEGG enrichment are shown in Figure 5, which shows that the DEGs were mainly enriched in the following: viral protein–cytokine and cytokine–receptor interactions; cytokine–cytokine-receptor interactions; IL-17 signaling pathway; phagosome; NF-κB signaling pathway; ABC transporter; TNF signaling pathway; Toll-like receptor signaling pathway; MAPK signaling pathway, etc.

**Figure 6:**
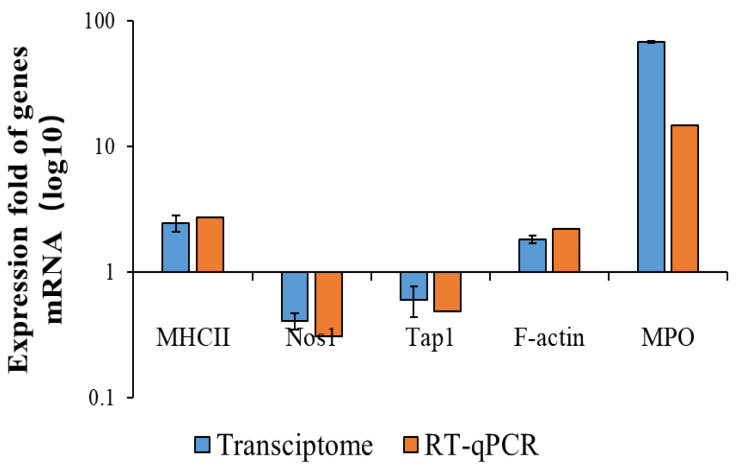
Comparison between transcriptome and RT-qPCR of some DEGs in the phagosome pathway.

The KEGG results showed that the pathways with the highest enrichment significance were viral-protein–cytokine and cytokine–receptor interactions and cytokine-–cytokine receptor interactions. This may have resulted from the LPS invading the host as a foreign antigen, stimulating the autoimmune mobilization of the host, and triggering the secretion of many cytokines, including chemokines from the CC subfamily, CXC subfamily, and interleukin family, as well as IL-6/12-like cytokines, IL1-like cytokines, IL17-like cytokines, etc. The activation of typical inflammatory pathways was further triggered by the over-secretion and dysregulation of these cytokines. These pathways include the NF-κB signaling pathway, Toll-like receptor signaling pathway, MAPK signaling pathway, etc. Furthermore, by analyzing the inflammatory pathways enriched in the EG/LPS group, we found that the COS caused multiple inflammatory pathways to respond, mainly because many receptors, such as Tlr4 and Cd14, were shared by two or more signaling pathways, and the COS had a significant effect on these upstream receptors’ expression. At the same time, the activation of these receptors can further trigger the activation of other non-inflammatory signaling pathways. For example, the RNA-seq data from the EG/LPS group showed that Tlr4 and c-lectin receptor, MR, in the Toll-like receptor signaling pathway were both upregulated. Both receptors further induced the enhancement of phagocytosis in the macrophages. The phagosome pathway showed that the F-actin expression on the cell membrane was significantly upregulated, indicating the addition of COS-enhanced phagocytosis. As it matured and acidified, the phagosome eventually become phagolysosome. In the phagolysosomes, myeloperoxidase (MPO) was significantly upregulated; MPO kills microorganisms in macrophages by catalyzing the oxidation of chloride ions to produce hypochlorous acid. Furthermore, MPO is hardly expressed in macrophages. We performed a RT-qPCR validation of several of the genes mentioned above. The results, which are shown in Figure 6, were generally consistent with the transcriptome results.

### 3.5. Pathways Validation Analysis

**Figure 7:**
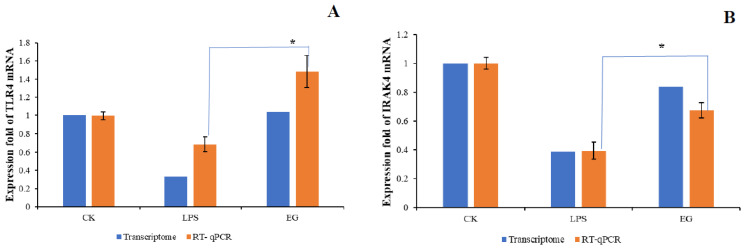

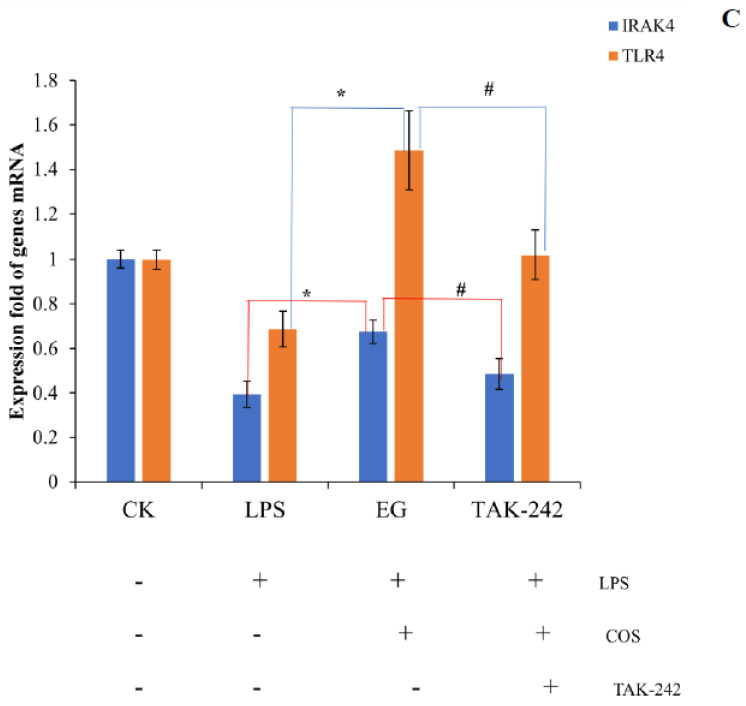
(**A**): Comparison between transcriptome and RT−qPCR of TLR4 in each group; (**B**): comparison between transcriptome and RT−qPCR of IRAK4 in each group; (**C**): expression levels of TLR4 and IRAK4 when the EG group was treated with TLR4 inhibitor, TAK−242. The * represents a significant difference compared with LPS group and # represents a significant difference compared with EG group.

Since the downstream of the Toll-like receptor signaling pathway overlaps with NF-κB, MAPK, and other inflammatory pathways, several nodal proteins were selected from the Toll-like receptor signaling pathway for the validation experiments. We validated the upstream–-downstream and key proteins to verify the RNA-seq results and found that COS plays a pleiotropic role in regulating LPS-induced inflammatory signaling pathways in macrophages. The results of the downstream inflammatory cytokines were shown in our previous report. This COS (DA = 12%) can significantly reduce the secretion of NO and IL-6,TNF-α and the expression of corresponding genes induced by LPS [29]. For the upstream, we first focused on two genes with significant differences: Tlr4 and Irak4. The Tlr4 gene belongs to the Toll-like receptor (TLR) family, which plays a fundamental role in pathogen recognition and innate immune activation. The Irak4 gene is one of the major regulators of the TLR/IL-1R downstream signaling pathway. When LPS was used to treat the cells alone, both the transcriptome and the RT-qPCR results showed that the LPS significantly reduced the expression levels of the Tlr4 and the Irak4. The EG group with the addition of COS was able to improve this effect, as shown in Figure 7A,B. Furthermore, the expression of both Tlr4 and Irak4 was suppressed in the EG group after the inhibition of Tlr4 using the Tlr4 inhibitor, Resatorvid (TAK-242), as shown in Figure 7C. These results indicate that the COS depends on the Tlr4 receptor to some extent, but not entirely. The Irak4 gene, which is downstream of Tlr4, is also regulated by COS.

**Figure 8:**
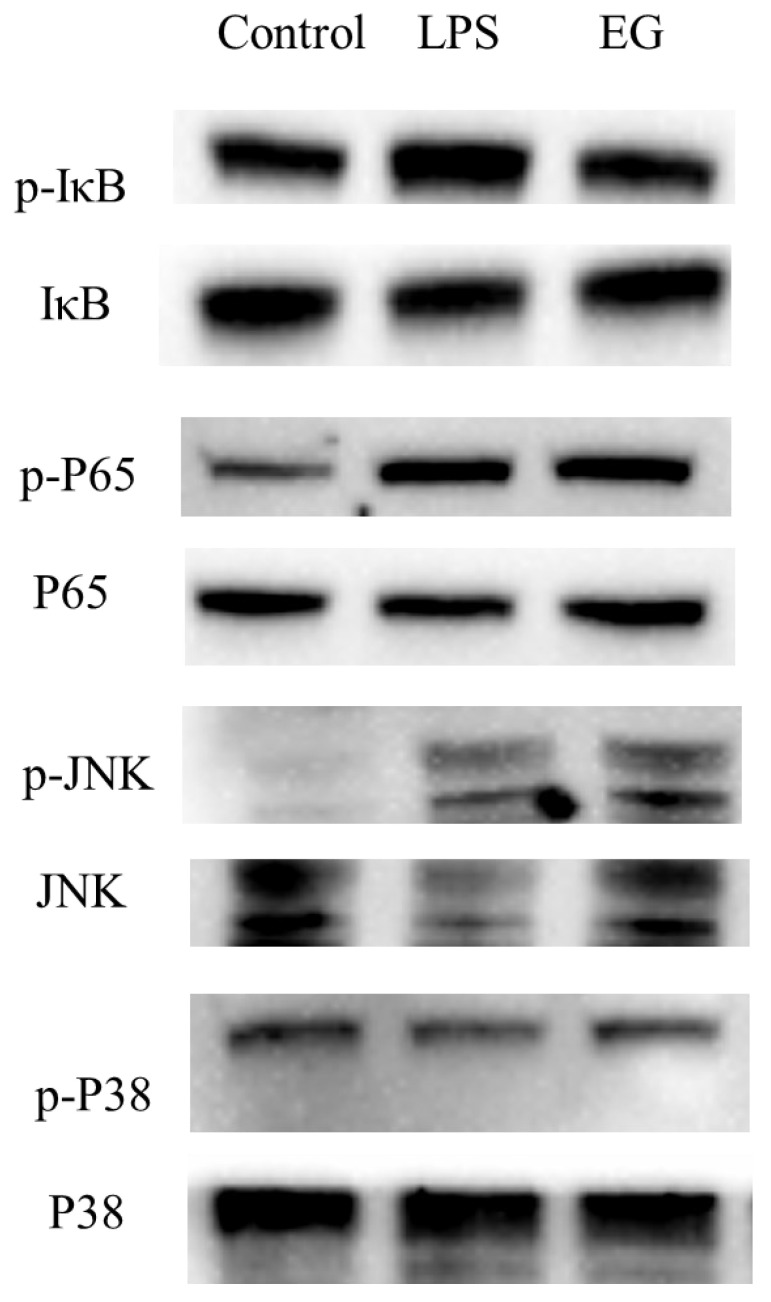
Expression and phosphorylation levels of key proteins on the Toll-like receptor pathway. The quantitative results of grayscale analysis are shown in Figure S2.

In addition to Tlr4, the addition of COS also significantly upregulated the expression of other receptors, such as Cd14, Tlr5, MR, etc., compared to the model group. The upregulation of these receptors activated some inflammatory pathways. However, the final phenomenon of the EG group showed its anti-inflammatory activity. It is crucial to study the expression of key proteins in these pathways. The downstream of the Toll-like receptor signaling pathway overlapped with other inflammatory pathways, such as NF-κB, MAPK, JAK-STAT, etc. There are many relevant reports on the occurrence of the anti-inflammatory effects of carbohydrates through the inhibition of the activation of the NF-κB signaling pathway. The NF-κB is a downstream nuclear transcription factor that directly regulates inflammation [24,30]. The NF-κB signaling pathway can be activated by TNF-𝛼, LPS, and other mediators. The receptor protein receives the stimulus and first activates IκB kinase (IKK). The IKK phosphorylates the serine at the regulatory site of the IκB subunit of the intracellular NF-κB-IκB complex, allowing the IκB subunit to be ubiquitinated and then degraded by the protease, thereby releasing the NF-κB dimer. The free NF-κB enters the nucleus and binds to genes with NF-κB binding sites to initiate the transcription process. Thus, the entry of NF-κB into the nucleus is a key step in the activation of inflammation [31]. Therefore, the phosphorylation of IκB and P65 was detected by the WB. As shown in Figure 8, the p-IκB/IκB ratio in the EG group was significantly lower than in the LPS group. For the phosphorylation of P65, there was also a slight decrease in the EG group compared to the LPS. These results suggested that COS can inhibit the activation of the NF-κB signaling pathway by inhibiting the phosphorylation of IκB and blocking NF-κB’s entry into the nucleus to some extent. In addition to the NF-κB signaling pathway, the MAPK signaling pathway is also highly enriched and overlaps with the Toll-like receptor signaling pathway and NF-κB signaling pathway. The MAPKs have several well-defined regulatory groups: ERK-1/2, JNK1/2/3, p38, and ERK5. In particular, JNK and p38 are quite complex and are related to inflammation, apoptosis, and growth. The ERK mainly governs cell growth and differentiation. Its upstream signal is the well-known Ras/Raf protein [32]. In the JNK family, activated JNK can bind to the amino-terminal regions of the transcription factors ATF2 and c-Jun to phosphorylate the active regions of the transcription factors. The activation of JNK means that its amino-terminal residues are phosphorylated. Once JNK is activated, it translocates to the nucleus to activate the inflammatory response. In the transcriptome results, the JNK was significantly upregulated in the EG group. Its phosphorylation was detected by the WB. As shown in Figure 8, the p-JNK/JNK in the LPS group was significantly higher than that in the EG and control groups. This result indicates that the addition of COS can significantly reduce the phosphorylation of JNK induced by LPS, which means that the anti-inflammatory activity of COS is related to its inhibitory effect on JNK phosphorylation. Since the expression of the MKK3/6 gene, the upstream gene of p38, was significantly upregulated in the EG/LPS, the MAPK/p38 signaling pathway was also examined. However, there were no significant differences between the EG and LPS groups. Furthermore, we inferred that the anti-inflammatory effect of the COS used in this experiment may be unrelated to the p38 signaling pathway.

## 4. Discussion

**Figure 9:**
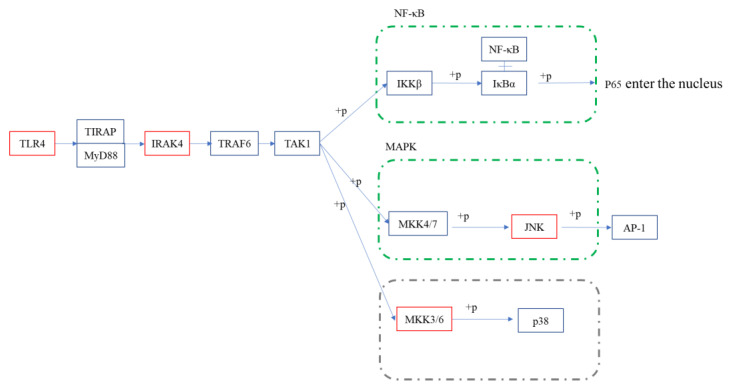
Schematic diagram of Toll-like receptor signaling pathways in the EG/LPS group. Red represents significant upregulation; p38 pathway is marked as gray because no significant change was detected.

Our previous report demonstrated that COS has good anti-inflammatory activity, mainly in inhibiting LPS-induced inflammatory-cytokine bursting, downregulating its mRNA expression, and that COS showed an obviously protective effect on endotoxemia mice. In order to systematically investigate its mechanism of anti-inflammatory activity, this paper reveals it, for the first time, through RNA-seq. By analyzing the KEGG results, COS was shown to exert pleiotropic modulation on the LPS-induced macrophages, mainly in the first-activated cytokines and cytokine receptors, followed by the dysregulation of these cytokines, triggering host autoimmunity, which in turn activated classical signaling pathways, such as NF-κB, MAPK, the Toll-like receptor signaling pathway and non-inflammatory pathways, the phagosome pathway, etc. The Toll-like receptor signaling pathway, overlapping with the NF-κB signaling pathway and the MAPK signaling pathway, was selected to verify these results. Based on the upstream expression of the Tlr4, Irak4, the phosphorylation of key proteins, and the expression of downstream cytokines (as shown in [29]), these signaling pathways were completely validated, and it was demonstrated that the anti-inflammatory activity of COS may be related to IκB and JNK, and not to p38 (schematic presentation in Figure 9).

In addition, we also compared the vast amount of data provided by the RNA-seq with previous studies. Jie Feng et al. demonstrated that the internalization of COS is mainly due to the presence of acetyl-glucosamine, and that mannose can competitively affect its internalization. They also proved that the internalization of COS depends on the mannose receptor to some extent [33]. Aotian Ouyang et al. systematically explored the mechanism of action of mannose receptors (MR) in COS internalization and concluded that MR and TLR4 act synergistically, although they used blunt-snout-bream macrophages [15]. Although MR has been studied more frequently, COS can still be internalized and show its activity after the inhibition of the MR, which is consistent with the increased expression of multiple receptors in the RNA-seq results. The expression of Tlr4, Tlr5, MR, and other receptors was significantly upregulated in the experimental group compared to the model group with the addition of LPS only. Lin Shi et al. also found the upregulation of Tlr4 expression in epithelial cells after COS addition [16]. However, the internalization of COS is only the first step, and the upregulation of receptor expression may activate the inflammatory pathway. However, the final cytokine-level results and mouse responses showed that the addition of COS exerted a significant anti-inflammatory effect. This was mainly due to the pleiotropic modulation of COS, which was not limited to the receptor level. According to the validation of the RNA-seq data, COS can significantly inhibit the phosphorylation of IκB and JNK. These results were also in line with those of previous studies, which reported that COS can exert anti-inflammatory activity by inhibiting the activation of the NF-κB signaling pathway [16,24,34]. Yu Li et al. even directly stated that COS can inhibit O-GlcNAcylation, an important step in the NF-κB signaling pathway [22]. However, in verifying the effect of COS on the MAPK signaling pathway, some studies reported that COS significantly reduced the phosphorylation of p38, which contrasted with our results [21,35]. This may have been due to the different degree of polymerization and acetyl content in the COS used. In summary, we speculate that the upregulation of the receptor expression was partly due to the internalization of the COS. The upregulation of the receptor mobilized the host autoimmunity to a certain extent. At the same time, the COS inhibited the entry of P65 and JNK into the nucleus by inhibiting the phosphorylation of the IκB and JNK. Eventually, lower levels of inflammation were restored to the macrophages.

## 5. Conclusions

This study suggested, for the first time, a pleiotropic modulation of COS in inflammatory signaling in LPS-induced macrophages at the level of RNA-seq and revealed its anti-inflammatory mechanism. The RNA-seq results showed that the experimental group treated with COS showed 2570 highly statistically significant genes compared with the model group. In total, 1239 of these genes were significantly upregulated and 1331 were significantly downregulated. These DEGs were mostly enriched in functions and pathways associated with inflammation and immunity. The KEGG enrichment results showed that the addition of COS upregulated the expression of many receptors, such as Cd14, Tlr4, Tlr5, etc., which in turn activate many inflammatory pathways, such as NF-κB, MAPK, toll-like receptor signaling pathways, etc. However, due to the ability of COS to significantly inhibit the phosphorylation of IκB, JNK, and P65, the macrophages were finally restored to a state of low inflammatory response. These results lay the foundation for the early application of COS as an anti-inflammatory drug.

## Figures and Tables

**Table 1:** The primers for RT-qPCR.

Gene	Primers
Irak4	F: AACAGACGGCCAGACATT R: CAACAGGTAGGTGACGCA
Map2k6	F: ACTGTGGATTGGTGGGTT R: CCTCAGAAAGGCAGGGT
Tlr4	F: CTTTGCTTCCTTGGTGTTG R: ATGATTCTCCTCTTCTTCACG
Cflar	F: CCGCAGGCTAACTTTCC R: CCCATCCCACTCAACAAC
Bcl-2	F: AAACCCTCCATCCTGTCC R: TCCTAAACCCTGCTTCCC
Fadd	F: TGCTCCATCTGGCTGTT R: GCATAGTCTGGGGAGTCAA
Il-1r	F: GTGCGGGACACTAAGGAG R: TTTACTGGGAGGCACAGC
Cd14	F: GACTCTGAATCCCACTCGGAGA R: TAGGAGCAAAGCCAGAGTTCCT
Cd40	F: GACTGCTTGCTGACCTTTG F: GACTGCTTGCTGACCTTTG
Ddit3	F: GAACAGTGGGCATCACCTC R: CAGTCCCCTCCTCAGCAT
H2-Dma	F: AATGCCCTGTGTGGTGTG R: GCCTGTGCTGTTCCAAGAT
Nos1	F: AAAACACAGGAGCAAGCG R: TGGTTGGGTCAGCAATCT
Mpo	F: CCCGCATTCCTTGTTTTC R: CTTCTCCCCATTCCATCG
Tap1	F: GTTGAGGAGCGGAGGTG R: TCAGTGTTGGCGTGAGGT

**Table 2:** Sample-data quality.

Sample	Raw Reads	Clean Reads	Clean Bases	Error (%)	Q20 (%) ^a^	Q30 (%) ^a^	GC (%) ^b^
CK-1	1.27 × 10^8^	1.27 × 10^8^	19.06 G	0.02	97.88	93.34	49.31
CK-2	94,739,692	94,739,544	14.19 G	0.02	97.96	93.43	49.05
CK-3	1.11 × 10^8^	1.11 × 10^8^	16.64 G	0.03	96.72	90.54	49.44
LPS-1	79,670,242	79,670,150	11.93 G	0.02	97.84	93.35	49.79
LPS-2	91,538,486	91,538,360	13.7 G	0.02	97.81	93.27	49.8
LPS-3	97,241,286	97,241,092	14.56 G	0.02	98.24	94.41	49.63
EG-1	99,173,740	99,173,666	14.84 G	0.03	97.14	91.57	50.25
EG-2	1.28 × 10^8^	1.28 × 10^8^	19.22 G	0.02	98.01	93.81	50.48
EG-3	1.2 × 10^8^	1.2 × 10^8^	18.01 G	0.03	97.7	92.97	50.75

^a^ Q20, Q30: Percentage of bases with base-mass fractions greater than 20 and 30 of the total bases. ^b^ GC: The total number of bases G and C as a percentage of the total number of bases.

## Data Availability

Not applicable.

## Supplementary Materials

The following supporting information can be downloaded at: https://www.mdpi.com/article/10.3390/polym15071613/s1, Figure S1: NO secretion by the cells used in this experiment. Figure S2: Quantitative analysis results of WB.
