# Supplementary material for: Pleiotropic Modulation of Chitooligosaccharides on Inflammatory Signaling in LPS-Induced Macrophages
Source: Polymers (Basel). 2023 Mar 23;15(7):1613. doi: 10.3390/polym15071613 (PMC10096960; doi:10.3390/polym15071613)
Supplement: Supplementary file 1 [file polymers-15-01613-s001.zip › polymers-2252933-supplementary materials.pdf]

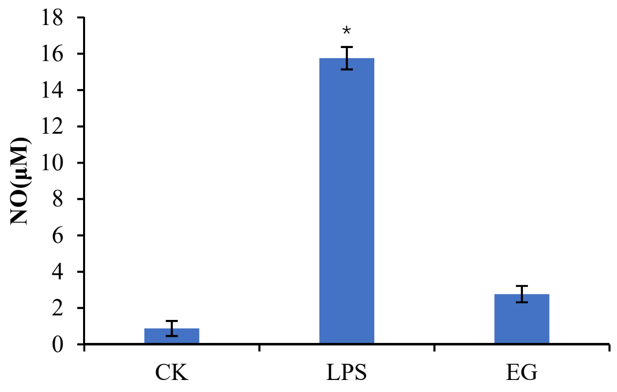

Fig.S1 NO secretion by the cells used in this experiment.

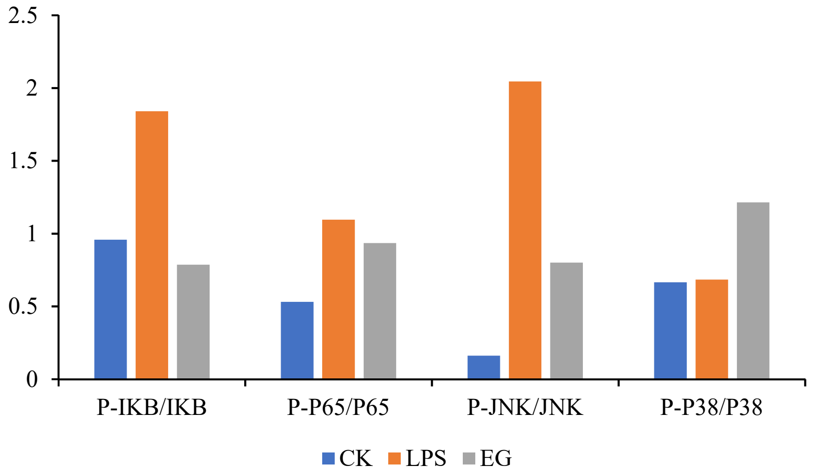

Fig.S2 Quantitative analysis results of WB
